# Differences in Recurrence Rate and *De Novo* Incontinence after Endoscopic Treatment of Vesicourethral Stenosis and Bladder Neck Stenosis

**DOI:** 10.3389/fsurg.2017.00044

**Published:** 2017-08-10

**Authors:** Jennifer Kranz, Philipp C. Reiss, Georg Salomon, Joachim Steffens, Margit Fisch, Clemens M. Rosenbaum

**Affiliations:** ^1^Department for Urology and Pediatric Urology, St. Antonius Hospital, Eschweiler, Germany; ^2^Department of Urology, University Medical Center Hamburg-Eppendorf, Hamburg, Germany; ^3^Martini Klinik Prostate Cancer Center, University Medical Center Hamburg-Eppendorf, Hamburg, Germany

**Keywords:** benign prostate hyperplasia, bladder neck stenosis, prostate cancer, transurethral resection, vesicourethral stenosis

## Abstract

**Objectives:**

The objective of this study was to compare the recurrence rate and *de novo* incontinence after endoscopic treatment of vesicourethral stenosis (VUS) after radical prostatectomy (RP) and for bladder neck stenosis (BNS) after transurethral resection of the prostate (TURP).

**Methods:**

Retrospective analysis of patients treated endoscopically for VUS after RP or for BNS after TURP at three German tertiary care centers between March 2009 and June 2016. Investigated endpoints were recurrence rate and *de novo* incontinence. Chi-squared tests and *t*-tests were used to model the differences between groups.

**Results:**

A total of 147 patients underwent endoscopic therapy for VUS (59.2%) or BNS (40.8%). Mean age was 68.3 years (range 44–86), mean follow-up 27.1 months (1–98). Mean time to recurrence after initial therapy was 23.9 months (1–156), mean time to recurrence after prior endoscopic therapy for VUS or BNS was 12.0 months (1–159). Patients treated for VUS underwent significantly more often radiotherapy prior to endoscopic treatment (33.3 vs. 13.3%; *p* = 0.006) and the recurrence rate was significantly higher (59.8 vs. 41.7%; *p* = 0.031). The overall success rate of TUR for VUS was 40.2%, success rate of TUR for BNS was 58.3%. TUR for BNS is significantly more successful (*p* = 0.031). The mean number of TUR for BNS vs. TUR for VUS in successful cases was 1.5 vs. 1.8, which was not significantly different. The rate of *de novo* incontinence was significantly higher in patients treated for VUS (13.8 vs. 1.7%; *p* = 0.011). After excluding those patients with radiotherapy prior to endoscopic treatment, the recurrence rate did not differ significantly between both groups (60.3% for VUS vs. 44.2% for BNS; *p* = 0.091), whereas the rate of *de novo* incontinence (13.8 for VUS vs. 0% for BNS; *p* = 0.005) stayed significantly higher in patients treated for VUS.

**Conclusion:**

Most patients with BNS are successfully treated endoscopically. In patients with VUS, the success rate is lower. Both stenoses differ with respect to *de novo* incontinence. Patients must be counseled regarding the increased risk of *de novo* incontinence after endoscopic treatment of VUS, independent of prior radiotherapy. Longer follow-up is warranted to address long-term outcomes.

## Introduction

Transurethral resection of the prostate (TURP) is still the most common and effective surgical method for the treatment of bladder outlet obstruction. Despite a significant reduction in the mortality rate, the morbidity of TURP is still about 15% and includes bleeding, urethral strictures, and the development of bladder neck stenosis (BNS) (1, 2). An extensive resection around the bladder neck may result in a stenosis, especially in small prostate glands (3). In the case of histologically proven prostatitis, the risk is even further increased (4). To prevent BNS, an additional incision of the internal sphincter could be an option for all smaller prostates. This incision causes a divergence of the internal fibers and thus an expansion of the bladder neck, but this is no guarantee for the prevention of a BNS (5). BNS occurs in about 0.3–9.2% of cases after TURP (6, 7), and no significant difference for BNS between bipolar vs. monopolar transurethral resection has been reported (8).

Besides radiotherapy, radical prostatectomy (RP) is an integral component of prostate cancer treatment especially for organ-confined tumors (9). A possible vulnerability after RP is the vesicourethral anastomosis, which is performed in single button technique or in a continuous manner. The incidence of vesicourethral stenosis (VUS) varies between 1.1 and 29% (10–16), depending on cancer treatment type. Rates of VUS after RP differ depending on the surgical procedure; the development of VUS is more common after open radical retropubic in comparison to robot-assisted RP (12, 17, 18) and more common after open radical retropubic in comparison to radical perineal prostatectomy (13). After salvage RP for locally recurrent prostate cancer after radiation therapy, rates of VUS are higher than those observed after standard RP (19, 20).

Anastomotic insufficiencies can arise primarily postoperatively or develop by shifting of the catheter when the patient is mobilized. It has been shown that the frequency of incontinence and the development of VUS increase after anastomotic leakage (21–23).

Treatment of VUS after RP and BNS after TURP and their recurrences are always a therapeutic challenge, even for experienced urologists. Treatment is usually initiated with an endoscopic approach commonly involving dilatation, direct vision of the internal urethrotomy, a bladder neck incision according to Turner Warwick, or transurethral resection of the bladder neck (11). Often, treatment is complicated by a combination of stenosis and urinary incontinence. However, different studies have shown a declining success rate after repeated transurethral surgery (24). Open surgical urethroplasty (25, 26) has been reported, as well as urinary diversion for recalcitrant stenoses (27).

In this study, we retrospectively analyzed patients treated endoscopically for VUS after RP or for BNS after TURP to compare recurrence rates and *de novo* incontinence. Our hypothesis is that the recurrence rates and rates of incontinence after therapy for either VUS or BNS are different.

## Materials and Methods

After obtaining Institutional Review Board approval, we retrospectively identified patients who underwent TUR for VUS or BNS from our institutional databases. We only included patients treated endoscopically for VUS after RP or for BNS after TURP. TUR was performed between March 2009 and June 2016. We excluded patients with laser treatment for VUS (*n* = 1) and patients with prior open simple prostatectomy (*n* = 2) due to small sample sizes.

Patients were interviewed by telephone using a standardized questionnaire (Supplementary Material) administered at the time of follow-up (FU). The questionnaire investigated previous urologic therapies (including radiation, endoscopic and open surgery), time to possible further therapy for the recurrence of VUS or BNS, type of further therapy and *de novo* incontinence after transurethral therapy.

Recurrence was determined as any need for further instrumentation such as catheterization, dilatation, internal urethrotomy, or open surgery. *De novo* incontinence was defined according to patient supplied information.

Descriptive statistics of categorical variables focused on frequencies and proportions. Means, ranges, and SDs are reported for continuously coded variables. Chi-squared tests and *t*-tests were used to model the differences between groups. All statistical analyses were performed using SPSS^®^ 20.0. The two-sided significance level was set at *p* < 0.05.

## Results

We identified 147 patients who underwent endoscopic therapy for VUS or BNS. Sixty patients (40.8%) underwent endoscopic treatment for BNS, and 87 patients (59.2%) underwent endoscopic treatment for VUS. The mean age of the entire cohort was 68.3 (range 44–86) years, mean FU was 27.1 (1–98) months. The mean time to recurrence after initial therapy was 23.9 (1–156) months, and the mean time to recurrence after prior endoscopic therapy for VUS or BNS was 12.0 (1–159) months. The mean time between prior therapy (RP, TURP or endoscopic treatment of VUS, or BNS) and recent therapy was 16.6 (1–159) months. The median number of prior endoscopic therapies for VUS or BNS was 1 (IQR 0–1). Tables 1 and 2 show the baseline characteristics of patients treated for VUS and for BNS.

**Table 1:** Patients treated for vesicourethral stenosis.

	*n* (%)
**Type of radical prostatectomy**	
Open	85 (97.7)
Laparoscopic/robot-assisted	2 (2.3)
**Radiation therapy**	
No	57 (65.5)
Low-dose BT	2 (2.3)
High-dose BT	1 (1.1)
Adjuvant EBRT	27 (31.0)
**Prior transurethral resection of the prostate**	
Yes	6 (6.9)
No	81 (93.1)

*BT, brachytherapy; EBRT, external beam radiation therapy*.

**Table 2:** Patients treated for bladder neck stenosis.

	*n* (%)
**Type of transurethral resection**	
Transurethral resection of the prostate	49 (81.7)
Laser (HoLEP)	6 (10.0)
Incision	5 (8.3)
**Radiation therapy**	
No	52 (86.7)
Low-dose BT	4 (6.7)
High-dose BT	2 (3.3)
EBRT	2 (3.3)

*BT, brachytherapy; EBRT, external beam radiation therapy*.

Table 3 demonstrates the differences between the two groups: patients treated for VUS underwent significantly more often radiation therapy prior endoscopic treatment (33.3 vs. 13.3%; *p* = 0.006), and the recurrence rate was significantly higher (59.8 vs. 41.7%; *p* = 0.031). Table 4 displays the success rate stratified by the number of procedures. The rate of *de novo* incontinence was significantly higher in patients treated for VUS (13.8 vs. 1.7%; *p* = 0.011), too. Further on, we analyzed how many patients were successfully treated endoscopically. TUR for VUS was successful in 50% of all cases, TUR for BNS was successful in 70% of all cases. Therefore, endoscopic treatment for BNS was significantly more successful in patients suffering from BNS (*p* = 0.022). The mean number of endoscopic treatments in successful cases was 1.5 (1–4) in TUR for BNS, 1.8 (1–6) in TUR for VUS. The number of endoscopic maneuvers before success did not differ significantly.

**Table 3:** Comparison of patients treated for VUS and BNS.

	VUS	BNS	*p*-value
Age [mean years (SD)]	67.7 (±8.48)	69.1 (±6.45)	n.s.
Time from initial therapy to stenosis [mean month (SD)]	28.14 (±31.28)	19.36 (±20.54)	n.s.
Time from prior endoscopic therapy to recurrence [mean month (SD)]	12.31 (±24.36)	11.31 (±16.63)	n.s.
Radiation therapy prior endoscopic treatment, *n* (%)	29 (33.3)	8 (13.3)	0.006
Qmax, mean ml (SD)	9.35 (±6.39)	6.24 (±3.47)	n.s.
PVR, mean ml (SD)	78.54 (±87.25)	126.63 (±31.4)	n.s.
Recurrence, *n* (%)	52 (59.8)	25 (41.7)	0.031
Time to recurrence [mean month (SD)]	5.85 (±7.27)	8.13 (±9.98)	n.s.
*De novo*-incontinence, *n* (%)	12 (13.8)	1 (1.7)	0.011

*BNS, bladder neck stenosis; VUS, vesicourethral anastomosis stenosis*.

**Table 4:** Success rates of patients treated by TUR for vesicourethral stenosis (VUS) and bladder neck stenosis (BNS), stratified by number of procedures.

	TUR for VUS		TUR for BNS		*p*-value
	*n* (successful treated/all treated pts)	%	*n* (successful treated/all treated pts)	%	
1. TUR	17/35	48.6	22/34	64.7	n.s.
2. TUR	12/26	46.2	9/17	52.9	n.s.
3. TUR	3/11	27.3	3/5	60.0	n.s.
≥4. TUR	3/15	20.0	1/4	25.0	n.s.

When comparing success rate of those who underwent radiotherapy (48.6%) vs. those who did not (47.3%), we saw no statistically significant effect. These results were confirmed in further analysis, in which we excluded patients with radiation therapy prior to endoscopic treatment: the recurrence rate did not differ significantly between groups (60.3% for VUS vs. 44.2% for BNS; *p* = 0.091). Using cox regression analysis radiotherapy did not turn out to be a significant predictive factor for recurrence.

*De novo* incontinence (13.8 vs. 0%; *p* = 0.005) stayed significantly higher in patients treated for VUS after excluding patients who had undergone radiation therapy.

## Discussion

This three-institutional analysis reports on the recurrence rate and the incidence of *de novo* incontinence after endoscopic treatment of VUS after RP and of BNS after TURP.

The retrospective data of 147 patients were analyzed (87 patients with VUS and 60 patients with BNS). VUS and BNS differed with respect to the recurrence rate and the *de novo* incontinence rate. We found higher recurrence rates and especially higher *de novo* incontinence rates in patients treated for VUS. The latter was independent from prior radiotherapy. Our hypothesis of different success and incontinence rates is thereby supported. This seems of utmost importance when counseling patients before therapy. Patients treated for VUS should be counseled about the burden of new onset incontinence especially after endoscopic therapy. There is a non-negligible risk of *de novo* incontinence after endoscopic incision of VUS (13.8%). As expected, only one patient with BNS suffered from *de novo* incontinence after multiple endoscopic surgeries. Similarly, low rates of incontinence for BNS were described in the obtainable literature (24).

Regarding BNS after TURP in our study cohort, 58.3% (35 of 60) were treated successfully, whereas 41.7% had a recurrence of the BNS. The mean time from initial therapy to stenosis was 19.36 months (±20.54); in the case of recurrence, the mean time from prior endoscopic therapy to recurrence was 11.31 months (±16.63). Comparing our data to the current literature, the results of endoscopic treatment for BNS are quite comparable. Pansadoro et al. presented data from 59 enrolled patients, with a mean age of 69 years and a median FU of 72 months (24). Of those patients, 51 (86%) were treated successfully. A total of three patients underwent a successful second endoscopic procedure, for a final success rate of 91% (54 of 59) (24). Recurrent strictures presented within 1 year after the operation at a median interval of 5 months (range 1–12) in their study (24), comparable with our own data.

Furthermore, Pansadoro et al. described some predisposing factors that have been suggested in the pathogenesis of type I strictures (BNS) after surgery on the prostate, including low average weight of the adenoma (12 g) compared with an overall median of 28 g and a subcervical adenoma with minimal residual urine (24). Due to missing data, e.g., weight of the adenoma, it is unfortunately not possible to state our own risk factors for the development of recurrences. These aspects will have to be considered in future research.

Consistent with the literature, patients suffering from VUS were treated successfully in 40.2% of cases (35 of 87) (24, 28). The mean time from initial therapy to stenosis was a bit longer than for BNS, but not significantly so.

The sole endoscopic treatment of recurrent BNS and VUS is generally associated with a high recurrence rate and is unsatisfactory for the patient as well as for the therapist due to frequent surgical interventions (29). Interesting combination treatments such as urethrotomy followed by HDR-brachytherapy (30) or internal urethrotomy followed by an adjuvant submucosal mitomycin injection (31) appear to have good short-term results, but experiences from larger series or long-term results are not available. Therefore, these treatment modalities cannot be recommended as a standard procedure.

Patients presenting with VUS and BNS should be informed about the outcomes of endoscopic therapy and the possibility of multiple procedures. As our results show, recurrent stenosis can be treated again endoscopically. However, the success rate after repeat endoscopic therapies decreases. Some studies have shown a declining success rate after repeated transurethral surgery (24, 28).

As our data show success rates of patients treated for VUS decrease with the number of endoscopic procedures (for third TUR only 27.3%), whereas 60% of patients with BNS benefited from another endoscopic surgery. A quite low success rate is anticipated for more than four endoscopic treatments for both BNS and VUS and should only be offered in an exceptional case (e.g., in cases of poor general health or comorbidities).

From our point of view, a stepwise approach for therapy starts with an endoscopic incision or resection of the narrowed anastomosis or bladder neck. In cases of recalcitrant and intractable VUS or BNS recurrences after repeated (for VUS, not more than three attempts) endoscopic therapies, we believe that patients require more invasive treatments. An open-operative, reconstructive procedure in a selected patient group with recurrent anastomotic strictures is preferable. Our preferred approach in cases of devastated bladder outlet obstruction is a transperineal reanastomosis for the treatment of highly recurrent VUS or a T-plasty or an YV-plasty for highly recurrent BNS (32, 33).

In most patients, in addition to obstructive micturition symptoms after frequent endoscopic interventions, *de novo* incontinence also develops; therefore, as a last resort treatment, a urinary diversion (27, 29, 34) is an option as well. This surgical procedure can be carried out without significant complications, the medium-term results are very good and suggest a good quality of life for patients (27, 29).

There are several limitations to our study. First, it was retrospective study and a relatively small number of patients were included. Further, there is no standardized measurement to objectify the recurrence of a stricture or incontinence. Moreover, a longer FU is warranted to address long-term outcomes. Nevertheless, we are the first to determine differences in treatment outcomes for two different conditions, i.e., VUS and BNS. To the best of our knowledge, this is the first study to determine this difference.

In conclusion, VUS and BNS are different entities and should be handled as such. In our institutional experience, most patients with BNS are treated successful endoscopically. In patients with VUS, the success rate is lower. Both stenoses differ with respect to *de novo* incontinence. Patients must be counseled about the increased risk of *de novo* incontinence after endoscopic treatment of VUS, independent of prior radiotherapy.

## Ethics Statement

We declare that, prior to the start of the study, all participating centers had attained ethical committee approval by their institutional review boards. Initial ethical committee approval was received in Hamburg (PV5205). The study was conducted in accordance with the Declaration of Helsinki (amendment by the 64th WMA General Assembly, Fortaleza, Brazil, October 2013).

## Author Contributions

JK: acquisition of data for the work, drafting the work, final approval of the version to be published, agreement to be accountable for all aspects of the work in ensuring that questions related to the accuracy or integrity of any part of the work are appropriately investigated and resolved. CR: substantial contributions to the conception of the work, revising the work critically for important intellectual content, final approval of the version to be published, agreement to be accountable for all aspects of the work in ensuring that questions related to the accuracy or integrity of any part of the work are appropriately investigated and resolved. GS, MF: substantial contributions to the design of the work, revising the work critically for important intellectual content, final approval of the version to be published, agreement to be accountable for all aspects of the work in ensuring that questions related to the accuracy or integrity of any part of the work are appropriately investigated and resolved. JS: analysis and interpretation of data for the work, revising the work critically for important intellectual content, final approval of the version to be published, agreement to be accountable for all aspects of the work in ensuring that questions related to the accuracy or integrity of any part of the work are appropriately investigated and resolved. CR: substantial contributions to the conception and design of the work; acquisition, analysis and interpretation of data for the work; drafting the work and revising it critically for important intellectual content; final approval of the version to be published; agreement to be accountable for all aspects of the work in ensuring that questions related to the accuracy or integrity of any part of the work are appropriately investigated and resolved.

## Conflict of Interest Statement

This study was conducted in the absence of any commercial or financial relationships that could be construed as a potential conflict of interest.

## Abbreviations

VUS, vesicourethral stenosis; RP, radical prostatectomy; BNS, bladder neck stenosis; TURP, transurethral resection of the prostate.
