# Supplementary material for: Differences in Recurrence Rate and De Novo Incontinence after Endoscopic Treatment of Vesicourethral Stenosis and Bladder Neck Stenosis
Source: Front Surg. 2017 Aug 10;4:44. doi: 10.3389/fsurg.2017.00044 (PMC5554361; doi:10.3389/fsurg.2017.00044)
Supplement: Supplementary file 1 [file data_sheet_1.docx]

**Appendix: Questionnaire**

Date of the last TUR:

Has a radical prostatectomy been performed before the above mentioned surgery?

- Yes
- No

If so, how was the surgical approach like?

- Open
- Laparoscopic
- Robot-assisted (DaVinci)

Has a radiation therapy been performed before the above mentioned surgery?

- Yes
- No

If so, what kind of radiation has been performed?

- LDR-brachytherapy (Seeds)
- HDR-brachytherapy (Afterloading)
- EBRT – as initial therapy
- EBRT – as adjuvant or salvage therapy

Has a simple prostatectomy been performed before the above mentioned surgery?

- Yes
- No

If so, how was the surgical approach like?

- TUR-P
- Lasertherapy (HoLEP, ThuLEP, Greenlight)
- Open simple prostatectomy

When was the first therapy before initial TUR-bladder neck/anastomosis?

If repeated TUR-bladder neck/anastomosis have been performed: When was the TUR-bladder neck/anastomosis performed?

Since the last surgery: Has there been a recurrence, defined as any further instrumentation such as catheterization etc.?

- Yes
- No

If so, how has the recurrence been treated?

- TUR
- Catheter
- Radical Cystectomy
- Perineal Reanastomosis/YV-Plasty/open surgery/Mitroffanof-Stoma

Since the last TUR: Did a new-onset incontinence occur?

- Yes
- No
